# Infant gut microbiota following cesarean section in the Middle East: a multidisciplinary expert consensus based on a targeted narrative review

**DOI:** 10.3389/fped.2026.1809889

**Published:** 2026-05-19

**Authors:** Wajeeh Aldekhail, Mohammed Al-Beltagi, Flavia Indrio, Abdulrahman M. Al-Nemri, Ahmed Tomerak, Ali Al Sawai, Fawaz Al-Refaee, Ghanem Al-Ghanem, Ibrahim Hosamuddin Rozi, Jubara Alallah, Junaid Muhib Khan, Khaled Abouhazima, Khaled Al-Mannaei, Khaled El-Atawi, Rola Sleiman, Mohammed Miqdady

**Affiliations:** 1 Department of Pediatric Gastroenterology & Hepatology, King Faisal Specialist Hospital & Research Centre, Riyadh, Saudi Arabia; 2 Department of Pediatrics, Faculty of Medicine, Tanta University, Tanta, Alghrabia, Egypt; 3 Department of Pediatrics, University Hospital, Arabian Gulf University, Manama, Bahrain; 4 Department of Experimental Medicine, University of Salento, Lecce, Italy; 5 College of Medicine, King Saud University, Riyadh, Saudi Arabia; 6 Department of Pediatric, King Saud University Medical City, Riyadh, Saudi Arabia; 7 Department of Neonatal Intensive Care Unit, Al Wakra Hospital, Hamad Medical Corporation, Doha, Qatar; 8 Department of Child Health, Royal Hospital, Ministry of Health, Muscat, Oman; 9 Department of Pediatrics, Al-Adan Hospital, Ministry of Health, Kuwait, Kuwait; 10 Department of Neonatal Perinatal Medicine, Adan Hospital, Ahmadi, Kuwait; 11 Department of Pediatrics, King Fahd Armed Forces Hospital, Jeddah, Saudi Arabia; 12 Department of Pediatrics, King Abdulaziz Medical City-Jeddah, Ministry of National Guard Health Affairs (MNGHA), Jeddah, Saudi Arabia; 13 College of Medicine-Jeddah, King Saud Bin Abdul Aziz University for Health Sciences (KSAU-HS), Jeddah, Saudi Arabia; 14 King Abdullah International Medical Research Centre (KAIMRC), Ministry of National Guard Health Affairs (MNGHA), Riyadh, Saudi Arabia; 15 Department of Neonatal Intensive Care Unit, Shakhbout Medical City/Mayo Clinics, Abu Dhabi, United Arab Emirates; 16 Division of Gastroenterology, Hepatology, and Nutrition, Sidra Medicine, Doha, Qatar; 17 Department of Paediatrics, Al Salam International Hospital, Dasma, Kuwait; 18 Department of Pediatrics, Latifa Women and Children Hospital, Dubai, United Arab Emirates; 19 Al Habib Medical Group, Al Rayyan Hospital, Riyadh, Saudi Arabia; 20 Pediatric Gastroenterology, Hepatology, and Nutrition, Sheikh Khalifa Medical City (SKMC), Abu Dhabi, United Arab Emirates; 21 Pediatric Gastroenterology, Hepatology, and Nutrition, College of Medicine and Health Sciences, Khalifa University, Abu Dhabi, United Arab Emirates

**Keywords:** breastfeeding (BF), cesarean section, early-life dysbiosis, first 1,000 days, gut microbiota, probiotics, term infant

## Abstract

**Background:**

Early-life dysbiosis associated with Cesarean section (C-section) delivery is increasingly recognized as a modifiable risk factor influencing short- and long-term health outcomes. This multidisciplinary expert consensus summarizes the clinical implications of C-section on infant gut microbiota. It proposes evidence-based strategies to mitigate these effects, with a focus on the critical window of the first 1,000 days of life.

**Methods:**

A multidisciplinary panel of 16 pediatricians, neonatologists, pediatric gastroenterologists, and nutrition experts conducted a targeted narrative review of the literature to inform a structured expert consensus process and participated in an online structured consensus process. Seventeen consensus statements were developed and validated through discussion, expert voting, and commentary, supported by a targeted review of current scientific evidence.

**Results:**

The expert panel reached consensus on the impact of C-section delivery on early microbiota composition and its clinical relevance, emphasizing that the rising prevalence of C-sections worldwide demands urgent attention. Experts unanimously emphasized the importance of exclusive breastfeeding as the primary strategy to support healthy microbiota development in infants born by cesarean section. When exclusive breastfeeding is not possible, evidence-based nutritional approaches, including selected prebiotics and probiotic strains with documented clinical efficacy, are recognized as promising alternatives for supporting microbial balance. Notably, the panel underscored that not all probiotics are equally effective and recommended shifting toward evidence-based strains shown to help restore gut dysbiosis in this population. Also, experts advocated for continuing microbiota-targeted support throughout the first 1,000 days of life, viewing this developmental window as a critical continuum rather than a limited early-life phase, while acknowledging the need for more long-term data. Additionally, education for healthcare professionals and parents about the long-term implications of C-section delivery was emphasized as a key enabler of the broader adoption of eubiosis-targeted strategies.

**Conclusions:**

Optimizing microbial colonization in infants born by cesarean section requires a multifaceted approach that prioritizes breastfeeding, supports judicious use of evidence-based nutritional interventions when needed, and emphasizes education and continuity of care across early life. By aligning clinical practice with emerging microbiome science, early-life interventions may reduce dysbiosis-associated risks and improve long-term health outcomes.

## 1 Introduction

The establishment of a diverse and balanced gut microbiota in the first 1,000 days of life—from conception through early childhood—is a critical determinant of long-term health, playing a central role in immune reprogramming, metabolic regulation, micronutrient absorption, and the physiological maintenance of epithelial integrity (1–3). Microbial colonization in early life is shaped by several perinatal factors, including mode of delivery, infant feeding practices, maternal-infant interactions, antibiotic exposure, and environmental exposures. Disruptions in microbial colonization during this window—particularly those associated with Cesarean section (C-section) delivery—have been linked to increased susceptibility to adverse health outcomes later in life (4, 5). Given this long-term importance of early normal microbial colonization, maintaining or restoring a healthy gut microbiome in early life has become a priority for healthcare professionals (HCPs).

According to the Developmental Origins of Health and Disease (DOHaD) framework, early environmental exposures during sensitive developmental windows, particularly the first 1,000 days, can permanently shape physiological systems and increase the susceptibility to non-communicable diseases (NCDs) (6). Among these influences, the mode of delivery is a key determinant of early microbial colonization. Vaginal delivery enables the vertical transmission of maternal vaginal and fecal microbiota, including *Bifidobacterium, Lactobacillus*, and *Bacteroides* species, crucial to neonatal immune and metabolic development (5, 7). In contrast, C-section delivery is associated with altered colonization by maternal skin- and hospital-associated microbes, such as *Staphylococcus, Klebsiella*, and other opportunistic species, as well as the delayed establishment of beneficial taxa, including short-chain fatty acid (SCFA)–producing bacteria (8, 9). This delayed colonization of the infant-type microbiota and reduced microbial diversity may persist for months to years and is commonly described as early-life dysbiosis (10, 11).

The altered microbiota seen in infants born by cesarean section represents a potential risk factor for increased risk of infections and for a range of NCDs, including allergic diseases, atopic dermatitis, food allergies, asthma, obesity, type 1 diabetes, and selected neurodevelopmental disorders (12–16) These associations are thought to be mediated, at least in part, by immune dysregulation and heightened inflammatory responses linked to disrupted early colonization (17, 18).

Critically, breastfeeding remains the most effective and physiologically appropriate intervention for the development of a healthy infant microbiota and for promoting immune tolerance (19, 20). However, when breastfeeding is not possible or is insufficient, nutritional approaches specifically tailored to infants born by cesarean section—such as infant formulae fortified with prebiotics, probiotics, or synbiotics—may help mitigate the impact of dysbiosis, particularly if designed to promote colonization by beneficial bacteria such as *Bifidobacterium* and *Lactobacillus* (21–24).

Furthermore, emerging evidence supports a continuity-based approach: interventions should not be limited to the neonatal period but extended across the full 1,000-day window to maximize long-term health benefits. The concept of “microbial care continuity” is emerging as an essential framework for proactively addressing the evolving needs of the infant microbiome and in alignment with developmental milestones (3, 25).

Despite growing evidence, current clinical practices and guideline recommendations related to microbiota support in infants born by cesarean section remain heterogeneous. To address this gap, Danone MENA convened a multidisciplinary expert panel of regional pediatricians, neonatologists, pediatric gastroenterologists, and nutrition specialists to develop evidence-informed, consensus-based guidance on how to best mitigate the microbiota-related health consequences of C-section delivery. This consensus document outlines the panel's key statements and practical recommendations, with an emphasis on breastfeeding as the foundational intervention, targeted nutrition, evidence-based use of synbiotics, and educational outreach.

## 2 Materials and methods

### 2.1 Expert selection process

To develop this multidisciplinary consensus, a panel of 16 experienced practicing clinicians and researchers from *Bahrain, Italy, Kuwait, Oman, Qatar, Saudi Arabia,* and *the United Arab Emirates* participated in an online consensus meeting. The panel included pediatricians, neonatologists, pediatric gastroenterologists, and nutrition experts with deep clinical experience in managing the health of infants born by cesarean section. Selection was based on recognized expertise in microbiome research, neonatal and infant nutrition, and regional insights into delivery practices. The diversity of the panel ensured a comprehensive understanding of the biological, clinical, and practical dimensions of early-life microbial modulation, with a focus on breastfeeding advocacy, precision in the use of prebiotics and probiotics, and long-term health considerations throughout the first 1,000 days. All experts participated voluntarily and contributed independent clinical judgment.

### 2.2 Evidence review and statement formulation

This work represents a multidisciplinary expert consensus statement supported by a targeted narrative literature review. Prior to the consensus meeting, Danone MENA, in collaboration with CCM International, conducted a targeted literature review to collate and summarize relevant scientific evidence for panel consideration. The targeted literature review included English-language, peer-reviewed studies focusing on human infants, particularly those delivered via C-section. Priority was given to randomized controlled trials, systematic reviews, and large observational cohort studies addressing microbiota composition, clinical outcomes, and nutritional interventions. Studies with insufficient methodological detail, non-human data, or a lack of relevance to predefined thematic areas were excluded. The compiled evidence focused on four predefined thematic areas:

1. The rising global and regional prevalence of C-section deliveries, which is above the recommended rates by the WHO.
2. The short- and long-term effects of C-section on infant gut microbiota composition and associated health outcomes.
3. Effective and evidence-supported nutritional strategies for supporting or restoring microbiota balance in infants born by cesarean section; and
4. The role of HCPs and parental education in adopting microbiota-supportive practices.

The expert panel was provided with a curated selection of relevant evidence identified through a targeted literature review. This approach aimed to prioritize high-quality, clinically relevant studies while ensuring the feasibility of the consensus process, rather than presenting the entire available literature. Draft consensus statements were developed in advance of the meeting based on current clinical practice, published evidence, and expert consultations. During statement formulation, specific attention was given to the following concepts:

- The importance of breastfeeding as the optimal and foundational intervention for microbiota development in all infants (19, 20),
- The efficacy of specific probiotic strains, when administered in combination with selectively utilized prebiotic substrates, in restoring microbiota profiles (e.g., *Bifidobacterium breve* M-16 V, *Lactobacillus rhamnosus* GG) (21, 24, 26),
- The emerging scientific consensus on the importance of continuity of microbial support throughout the first 1,000 days, rather than limiting interventions to the neonatal period (3, 25).

These topics were selected to ensure the final recommendations reflected the evolving understanding that early-life dysbiosis is both modifiable and time-sensitive, and that targeted strategies must go beyond one-size-fits-all solutions.

### 2.3 Quantification of consensus and voting process among experts

Prior to the live consensus meeting, panel members independently reviewed and voted on each draft statement, using a three-point scale: “disagree,” “partially agree,” and “fully agree”. Out of the 16 experts, one member, the panel chair, did not participate in the voting to maintain neutrality. These votes provided a baseline for identifying areas of consensus and divergence. The percentage of experts selecting each option was calculated.

During the structured virtual meeting, the panel engaged in evidence-guided discussions of each statement, with opportunities to revise content for clarity, accuracy, or scope. The experts were encouraged to discuss each statement extensively within the group and amend it accordingly. At the end of the discussion, each panelist could reconsider their original assessment or provide their final comments. Finally, agreement and discussion among the experts were also recorded.

**Figure 1:**
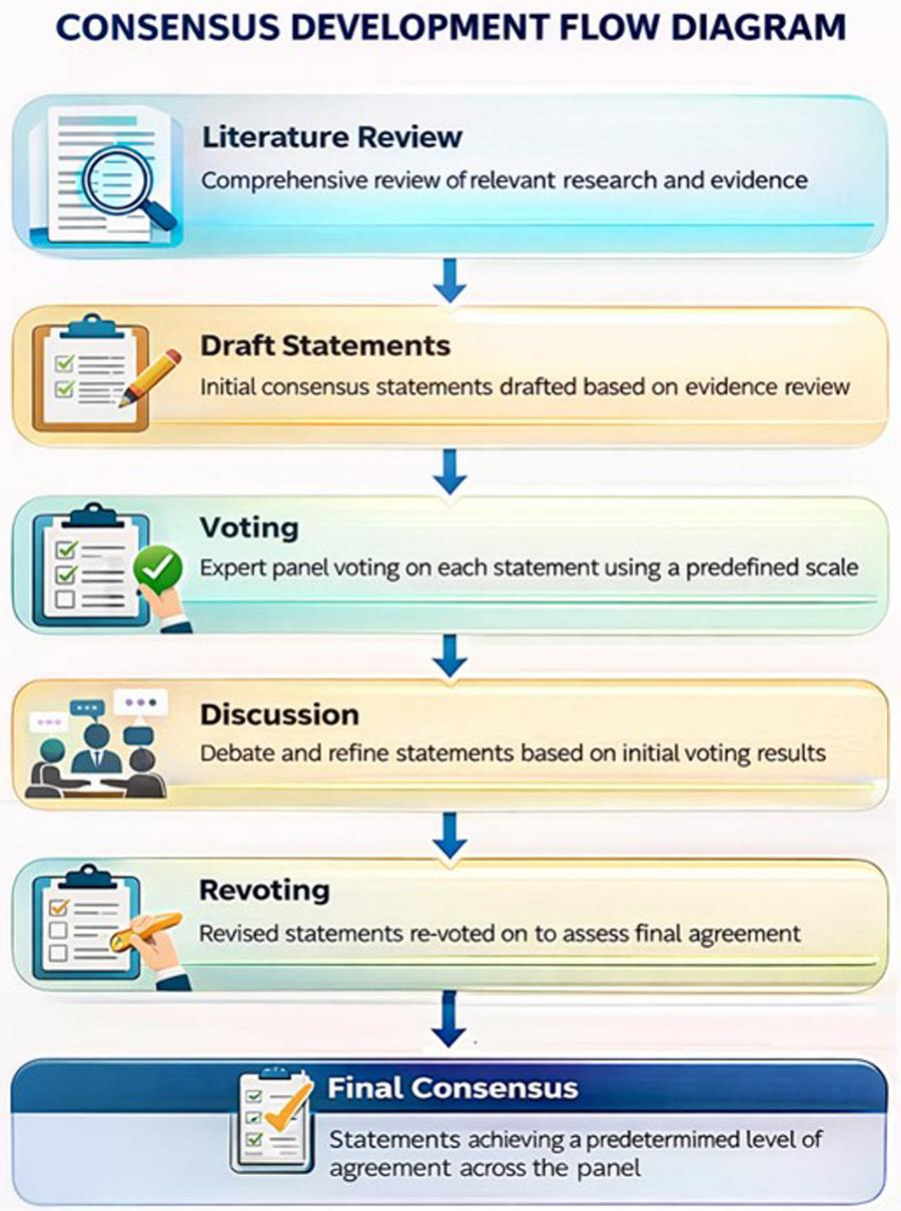
Structured consensus development process for generating multidisciplinary expert recommendations on infant gut microbiota following cesarean section. This figure illustrates the stepwise methodology used to develop the expert consensus. A targeted literature review was first conducted to identify relevant clinical and scientific evidence. Based on this evidence, draft statements were formulated and subjected to an initial round of voting by the expert panel using a predefined agreement threshold. Statements that did not reach consensus underwent structured discussion to refine wording and interpretation. Revised statements were then reassessed through a second round of voting (revoting). Final consensus was defined by achieving a predetermined level of agreement among panel members. This iterative process ensured that recommendations were evidence-informed, transparent, and reflective of multidisciplinary expertise.

Following discussion and revision, a revote was conducted. A predefined agreement threshold of ≥75% was used to define consensus. This process ensured that the final expert recommendations reflected a high level of agreement, clinical applicability, and credibility for understanding the long-term effects of C-section on gut microbiota composition and existing mitigating strategies. Final recommendations were shared with all panelists for endorsement and represent a synthesis of scientific evidence and expert judgment, emphasizing breastfeeding as the first-line intervention, cautious use of synbiotics when appropriate, and the principle of continuity of microbiota care throughout early life. Figure 1 shows the consensus development flow diagram.

The methodology aligns with established approaches to consensus-based recommendations, in which targeted evidence synthesis is integrated with expert interpretation to address clinically relevant questions in areas where high-quality randomized evidence may be limited. Specific probiotic strains or synbiotic formulations mentioned in this manuscript are provided as illustrative examples based on available clinical evidence and should not be interpreted as exclusive recommendations. Other formulations with comparable composition and supporting clinical data may also be appropriate.

## 3 Results

### 3.1 C-section prevalence globally and in the Middle East

The expert panel reviewed current epidemiological data demonstrating a sustained global increase in C-section delivery rates, a trend considered increasingly concerning for infant health and microbiota development. According to the World Health Organization (WHO), global C-section rates surged from approximately 7% in 1990 to 21% in 2021, with projections suggesting a further rise to nearly 29% by 2030 (27). These figures exceed the WHO-recommended threshold of 10%–15%, beyond which no additional reductions in maternal or neonatal mortality are typically observed (28).

This trend is particularly prominent across the Middle East, where C-section rates in several countries consistently exceed global averages. The panel reviewed regional data indicating that:

- Egypt, Iran, and Turkey regularly reported rates exceeding 40% (27, 29).
- In the United Arab Emirates, the national prevalence of C-sections reached 42.5% in 2021 (30), with notable inter-emirate variation, including rates of 15.4% in Dubai (31) in 2017 and 30.2% in Abu Dhabi (32).
- Kuwait reported a rate of 35.3% in 2023 (33).
- Lebanon documented a rate of 49.5% in 2019 (34).
- Oman reported 23.6% in 2022 (35).
- Qatar experienced an increase from 16.3% in 1998 to 29.8% by 2013 (36).
- KSA-based studies reported rising C-section rates ranging from approximately 19% in public hospitals to over 30% in urban settings (37, 38).

The panel unanimously agreed that this rising prevalence represents a global public health concern, especially in the context of microbiota-mediated health outcomes. Such variability across countries was considered to reflect a multifactorial interplay of healthcare system infrastructure, medical practice patterns, socioeconomic factors, maternal preferences, and cultural influences. Nonetheless, a consistent regional pattern of rising C-section utilization was identified, including procedures performed in the absence of clear medical indications.

The panel emphasized that these regional trends amplify the need for:

- Clear, evidence-informed clinical guidelines to promote microbiota-conscious C-section practices.
- Targeted education for both clinicians and parents regarding potential long-term health considerations.
- And the integration of microbiota-supportive interventions, such as breastfeeding promotion and, when breastfeeding is not feasible, the judicious use of evidence-based synbiotics.

Ultimately, the panel reached complete consensus that increasing global and Middle Eastern C-section rates pose substantial challenges to neonatal gut health, underscoring the necessity of proactive, evidence-based strategies to safeguard infant microbiota development (Table 1).

**Table 1:** Expert consensus on C-section prevalence globally and in the Middle East.

No.	Statement	Disagree	Partially agree	Agree
1	According to new research from the World Health Organization (WHO), C-section prevalence continues to rise globally	0%	0%	100%
2	Related to global prevalence trends and local evidence, the overall prevalence of C-section in the Middle East region is clearly increasing	0%	6.67%	93.33%

Percentages are based on responses from 15 voting panel members. The full panel included 16 experts; the chairperson abstained from voting to maintain neutrality.

### 3.2 Early-life gut microbiome, C-section delivery, and its impact

The expert panel unanimously agreed that the mode of delivery is a major determinant of early-life gut microbiome, a relationship that is supported by a substantial body of clinical, epidemiological, and mechanistic evidence. Consequently, the first four of the evaluated statements of the present segment (Table 2), which addressed the distinct microbial composition in infants born by cesarean section, the reduced microbial diversity and dysbiosis, the associated long-term health risks, and the colonization by hospital-associated bacteria, were reinstated during the meeting, not merely as consensus-based statements, but as evidence-supported associations.

**Table 2:** Expert consensus on early-life gut microbiome, C-section delivery and its impact.

No.	Statement	Disagree	Partially agree	Agree
3	The gut microbiome of a C-section-delivered baby differs from that of a infants born by vaginal delivery. The mode of delivery significantly impacts the composition of an infant's gut microbiome	0%	0%	100%
4	C-section delivery appeared to decrease the diversity of gut microbiota in neonates, leading to dysbiosis	0%	0%	100%
5	The compromised gut microbiota in C-section born babies is potentially linked to long-term health implications like immune-related disorders, obesity, and allergies, such as asthma	0%	0%	100%
6	Based on evidence, newborns delivered by C-section tend to harbor in their gut potential pathogenic bacteria commonly found in hospitals (e.g., *Enterococcus* and *Klebsiella*) and mother's skin surface, and have inefficient levels of beneficial bacteria, as found in infants born by vaginal delivery (e.g., *Bacteroides* and *Bifidobacterium* species)	0%	6.67%	93.33%
7	*Bifidobacterium* is considered a crucial taxonomic group in the early stages of intestinal microbiome development, being a predominant bacterium and among the primary colonizers in infants born by vaginal delivery.	0%	0%	100%
8	The microbiota plays a fundamental role in the induction, training, and function of the host immune system	0%	0%	100%
9	Current evidence suggests that early life, defined as the first 1,000 days, provides an opportunity for modulating the gut microbiota to promote long-term health.	0%	0%	100%
10	Studies suggest that C-section might be a risk factor for developing functional gastrointestinal disorders (e.g., functional constipation, infantile colics…) due to the altered gut microbiota also called dysbiosis.	0%	6.67%	93.33%
11	Based on evidence, C-section born babies have higher risk of infections	0%	13.33%	86.67%
12	C-section is associated with delayed colonization of the *Bacteroidetes* phylum, and reduced diversity of total microbiota in the first five years after birth	0%	6.67%	93.33%

Percentages are based on responses from 15 voting panel members. The full panel included 16 experts; the chairperson abstained from voting to maintain neutrality.

More specifically, all voting panel members agreed (100%) that the gut microbiome of an infant born by cesarean section differs significantly from that of an infant born by vaginal delivery. In particular, C-section delivery was consistently associated with reduced gut microbial diversity in neonates, a hallmark commonly described as a marker of dysbiosis (39). Importantly, C-section delivery is associated with disrupted vertical transmission of maternal microbes and delayed colonization by beneficial species, predisposing infants to dysbiosis and its downstream effects. As such, the growing rate of surgical deliveries highlights the urgency of implementing preventive and restorative microbiota strategies, particularly in vulnerable populations.

Vaginal delivery is typically associated with early colonization by maternal vaginal and intestinal microbes, such as *Lactobacillus* and *Bifidobacterium* species, which have been shown to support gut and immune health by aiding digestion, producing SCFAs, and promoting immune tolerance (4, 40). By contrast, C-section delivery is more frequently associated with colonization by skin-associated and environmental microbes from hospital operating rooms (e.g., *Staphylococcus*, *Corynebacterium*), whose presence is considered a marker of dysbiosis (17, 20, 41, 42). The experts noted that this altered microbial profile is shaped during the neonatal period and can persist for several months, potentially impairing immune system maturation and influencing allergic, autoimmune, and metabolic outcomes in later life. On the other hand, they also acknowledged evidence that microbial diversity may partially normalize over time, particularly with breastfeeding or other supportive exposures. However, the panel noted that despite this potential recovery, the disruption occurs during a critical window of microbiota, immune, and gut development, raising concerns about possible long-term health implications.

To this end, the discussion next focused on long-term health issues that can arise from the altered gut microbiota observed in infants born by cesarean section, a fact supported by a large body of epidemiological and mechanistic evidence (43). Early disruptions in microbial exposure, such as C-section delivery or antibiotic administration, may result in an overreactive or underdeveloped immune system, leading to lasting effects (44). These can include immune-mediated, allergic, and metabolic conditions, such as asthma, atopic disease, obesity, and type 1 diabetes (12, 14, 43). Still, the panel cautioned that such associations can be influenced by multiple confounding factors, including host genetics, infant feeding practices, and antibiotic exposure (17, 20, 41). Accordingly, causality cannot be inferred from observational data alone. Furthermore, on a related point which had already briefly been discussed, 93.33% of the panel (Table 2) supported the statement that C-section newborns tend to harbor potentially pathogenic bacteria such as *Enterococcus* and *Klebsiella*, commonly acquired from hospital environments and maternal skin, thus reflecting the surgical mode of delivery and the higher likelihood of early antibiotic exposure, while having reduced abundance of taxa commonly observed in infants born by vaginal delivery (e.g., *Bacteroides* and *Bifidobacterium* spp.) (17, 41).

Following, the panel focused on the importance of *Bifidobacterium* as a predominant early colonizer in infants born by vaginal delivery who had breastfeeding. This genus contributes to the utilization of human milk oligosaccharides (HMOs), SCFA production, gut barrier integrity, and immune modulation; thus, *Bifidobacterium* was noted as crucial to the establishment of a protective and balanced gut ecosystem (19). Reduced colonization in infants born by cesarean section was acknowledged as a plausible contributor to various adverse health outcomes. Overall, the gut microbiota was unanimously (100%) agreed to play a fundamental role in the induction, training, and function of the host immune system. Evidence reviewed during the meeting demonstrated that early microbial exposures support the maturation of immune cells, help balance inflammatory responses, and promote tolerance to non-harmful antigens (18). From a mechanistic perspective, commensal microbes influence the development of gut-associated lymphoid tissue (GALT) and the production of immunoglobulin A (IgA), both of which are essential for immune homeostasis (45). Consequently, the panel emphasized that disruptions to this process may predispose infants to immune-mediated diseases. Critically, the panel emphasized that early life—particularly the first 1,000 days from conception to age two—represents a critical and time-sensitive window of opportunity for microbiota modulation to promote long-term health (25). Intervening during this period with breastfeeding, or when necessary, tailored nutritional strategies (e.g., prebiotics, probiotics, synbiotics), is essential for restoring microbial balance and promoting long-term health.

With respect to functional gastrointestinal disorders, a strong majority of experts (93.33%) agreed that C-section delivery may be associated with an increased risk of conditions such as functional constipation, infantile colic, and irritable bowel syndrome (IBS), although the evidence base was described as heterogeneous. Specifically, a large cohort study (JECS) reported a slight increase in functional constipation by age 3 among children born by C-section, yet infant colic has shown no consistent association with delivery mode (46, 47). These conditions may involve alterations in gut-brain axis signaling and immune regulation, potentially linked to early-life microbial composition (48). Reduced abundance of beneficial bacteria such as *Bifidobacteria* and *Lactobacilli* in C-section-born infants was discussed as a possible contributing factor (3, 47, 49). On a broader scale, the panel reached 86.67% agreement with the statement that infants born by cesarean section face an increased risk of infections (15, 50), with 13.33% partially agreeing, noting that the term “infection” encompasses heterogeneous outcomes (Table 2). The panel acknowledged moderate, consistent evidence suggesting a small but statistically significant increase of infections (especially respiratory and gastrointestinal) among C-section–born children, requiring hospitalization during early childhood (51). The association diminishes with age and may be influenced by many confounding factors (feeding mode, postnatal exposures, hygiene) (51, 52).

Finally, 93.33% of experts agreed that C-section delivery is associated with delayed colonization by the Bacteroidetes phylum and reduced total microbial diversity, which may persist for up to 5 years after birth 11,39. While recovery timelines vary depending on population, feeding, and antibiotic administration—with some studies noting recovery within 6–12 months (53, 54), given that *Bacteroidetes* play a key role in carbohydrate metabolism and the production of health-promoting SCFAs, such alterations in microbial colonization patterns may have lasting effects on gut and metabolic health (55, 56). Early interventions, particularly prolonged breastfeeding and, where appropriate, targeted nutritional strategies such as clinically supported microbiota-restoring formulas, were considered potential modifiers of these trajectories.

### 3.3 Nutritional strategies for restoring gut microbiota

The expert discussion reviewed nutritional strategies to support the development and restoration of a balanced gut microbiota in infants born via C-section. There was unanimous agreement that exclusive breastfeeding remains the optimal and most effective strategy for establishing a healthy gut microbiome, including in C-section–born infants. Breast milk promotes colonization by beneficial taxa, particularly *Bifidobacterium* species. It contains bioactive components such as human milk oligosaccharides (HMOs), present at a dosage of 12–15 g/L, with more than 1,000 structures of varying chain lengths, which provide the quantitative and structural diversity that selectively favors the establishment of beneficial microbes (57, 58). Moreover, secretory IgA and antimicrobial peptides, which are also present in breast milk, contribute to gut and immune development (19, 20). On this basis, the panel reaffirmed breastfeeding as the foundational microbiota-supportive intervention whenever possible.

In situations where exclusive breastfeeding is not feasible or sufficient, the panel emphasized the importance of using nutritional solutions specifically tailored to the needs of C-section–born infants. These solutions should be supported by robust clinical evidence and may include infant formulae supplemented with prebiotics, probiotics, or synbiotics that have demonstrated clinical efficacy in promoting beneficial microbial colonization and mitigating features of early-life dysbiosis, thereby mimicking the quantity, diversity, and functionality of breastmilk components. For example, probiotic strains such as *Bifidobacterium* and Lactobacillus, as well as symbiotic formulations combining prebiotics and probiotics, have been shown to mimic some of the microbial benefits of breast milk, thereby helping to reduce the risk of dysbiosis and supporting gastrointestinal and immune maturation (21, 24, 59). Notably, some experts have suggested that this time frame could be expanded to encompass the entire first 1,000 days of life, which defines early life, as this developmental period is a critical window for shaping microbiota composition and programming the immune system. In agreement with this suggestion, the panel unanimously agreed that microbiota-targeted nutritional interventions may be relevant beyond infancy and throughout the entire first 1,000 days of life, not just the first year, to achieve long-term health benefits. In conclusion, to the discussion of the above statements, the experts highlighted the importance of strategies such as prebiotic, probiotic, and synbiotic supplementation (1, 2). Importantly, the panel stressed that not all probiotics are equal and strongly recommended using only strains with documented efficacy in C-section populations to support infant gut health. Furthermore, given the role of prebiotics in modulating the infant gut microbiota, future consensus efforts could consider selection criteria such as compositional diversity, functional properties, and dosage. In this context, prebiotic blends such as short-chain galacto-oligosaccharides (scGOS) and long-chain fructo-oligosaccharides (lcFOS), provided at 8 g/L with approximately 100 structures and a short: long ratio of 9:1, have been recognized to closely resemble HMOs in complexity, diversity, and functionality, making them particularly relevant for infant nutrition (60).

The panel strongly endorsed a synergistic approach combining prebiotics and probiotics to help reestablish gut microbial balance in infants born by cesarean section. Specifically, 93.33% of experts agreed, and 6.67% expressed partial agreement. More specifically, carefully selected prebiotics, such as the scGOS/lcFOS blend, serve as substrates that selectively feed beneficial bacteria, while probiotic strains like *Bifidobacterium breve* M-16V contribute directly to colonization. Clinical evidence supports the use of combined supplementation in modulating the early gut environment of infants born by cesarean section (21, 24, 61). Notably, the panel highlighted recent studies showing that early supplementation with a specific synbiotic blend, comprising scGOS/lcFOS, and *B. breve* M-16V, may compensate for the delayed colonization of Bifidobacteria commonly observed in infants born by cesarean section (21, 24, 26). This intervention has been associated with a reduction in the abundance of potentially harmful species such as *Clostridium difficile*, enhanced acetate production, which is a beneficial SCFA, and the acidification of the intestinal environment—features consistent with a microbiota profile more commonly observed in infants born with vaginal delivery (21, 24, 26, 62) This early synbiotic blend supplementation with scGOS/lcFOS and *B. breve* M16-V in infants born by cesarean section allows a fast colonization by Bifidobacteria from the first days of life, which may accelerate microbial maturation and provide immune-modulating benefits (21, 24, 26). To this end, such supplementation contributes to creating a gut environment that more closely resembles that of infants born by vaginal delivery. Additionally, the panel advocated for a continuity-of-care model, in which microbiota-targeted interventions are extended beyond infancy and throughout the first 1,000 days of life (3, 25). This approach recognizes the ongoing development of the gut microbiota and immune system well into toddlerhood and aligns with the DOHaD principles.

In line with earlier discussions on immune development, the panel unanimously agreed (100%) that restoring the compromised microbiota in infants born by cesarean section may support immune tolerance, in turn potentially lowering the incidence of skin disorders (Table 3). Discussion focused on evidence linking early-life dysbiosis to altered immune regulation and increased susceptibility to allergic disease (63–65) The panel unanimously agreed that by reestablishing a more balanced microbial community, nutritional strategies may support immune tolerance, strengthen mucosal defenses, and reduce inflammatory responses, potentially lowering the incidence or severity of skin disorders (66).

**Table 3:** Expert consensus on nutritional strategies for restoring gut microbiota.

No.	Statement	Disagree	Partially agree	Agree
13	If exclusive breastfeeding is not possible, an evidence-based nutritional solution tailored for C-section-born babies should be considered to modify gut microbiota composition during the first year.	0%	0%	100%
14	Some interventions to restore a perturbed microbial community might be efficient throughout the first 1,000 days	0%	0%	100%
15	A combination of specific prebiotics and probiotics is supported by clinical evidence to help restore the gut microbiota in infants, and it may be a good option for nutritional supplementation in babies born by cesarean section.	0%	6.67%	93.33%
16	Restoring the compromised gut microbiota in C-section born babies might improve some health outcomes, leading to reduced skin disorders (ex, atopic dermatitis, eczema)	0%	0%	100%

Percentages are based on responses from 15 voting panel members. The full panel included 16 experts; the chairperson abstained from voting to maintain neutrality.

Overall, the expert panel endorsed early and targeted nutritional interventions, which can act synergistically (e.g., breastfeeding as the first-line strategy, synbiotic-enriched formulas when exclusive breastfeeding is not possible, and the targeted use of validated combination of prebiotics and probiotic strains), as key tools for modulating the infant microbiome in vulnerable populations, such as infants born by cesarean section, to support beneficial microbial colonization, immune maturation, and long-term health. Emphasis was placed on adopting a longitudinal perspective that addresses microbial development throughout the full 1,000-day window of opportunity.

### 3.4 The role of education

The expert panel unanimously agreed that education represents a central and enabling component of strategies aimed at addressing the potential long-term health implications associated with C-section–related dysbiosis (Table 4). Providing HCPs and parents with evidence-based information was considered essential to support the appropriate adoption of microbiota-supportive feeding practices during the critical early-life window. A key concern identified during the panel discussion was the persistence of misconceptions regarding microbiota-targeted interventions, particularly the assumption that all probiotic products confer equivalent benefits. The panel emphasized that educational initiatives should clearly communicate the strain-specific nature of probiotic effects and underscore that only clinically validated strains—such as *Bifidobacterium breve* M-16V—have demonstrated efficacy in restoring microbiota balance in this population when combined with scGOS/lcFOS.

**Table 4:** Expert consensus on the role of education.

No.	Statement	Disagree	Partially agree	Agree
17	Educating parents and HCPs on the short- and long-term health impact of C-section on infants is vital to increase awareness and improve adaptation of tailored nutritional strategies	0%	0%	100%

Percentages are based on responses from 15 voting panel members. The full panel included 16 experts; the chairperson abstained from voting to maintain neutrality.

**Figure 2:**
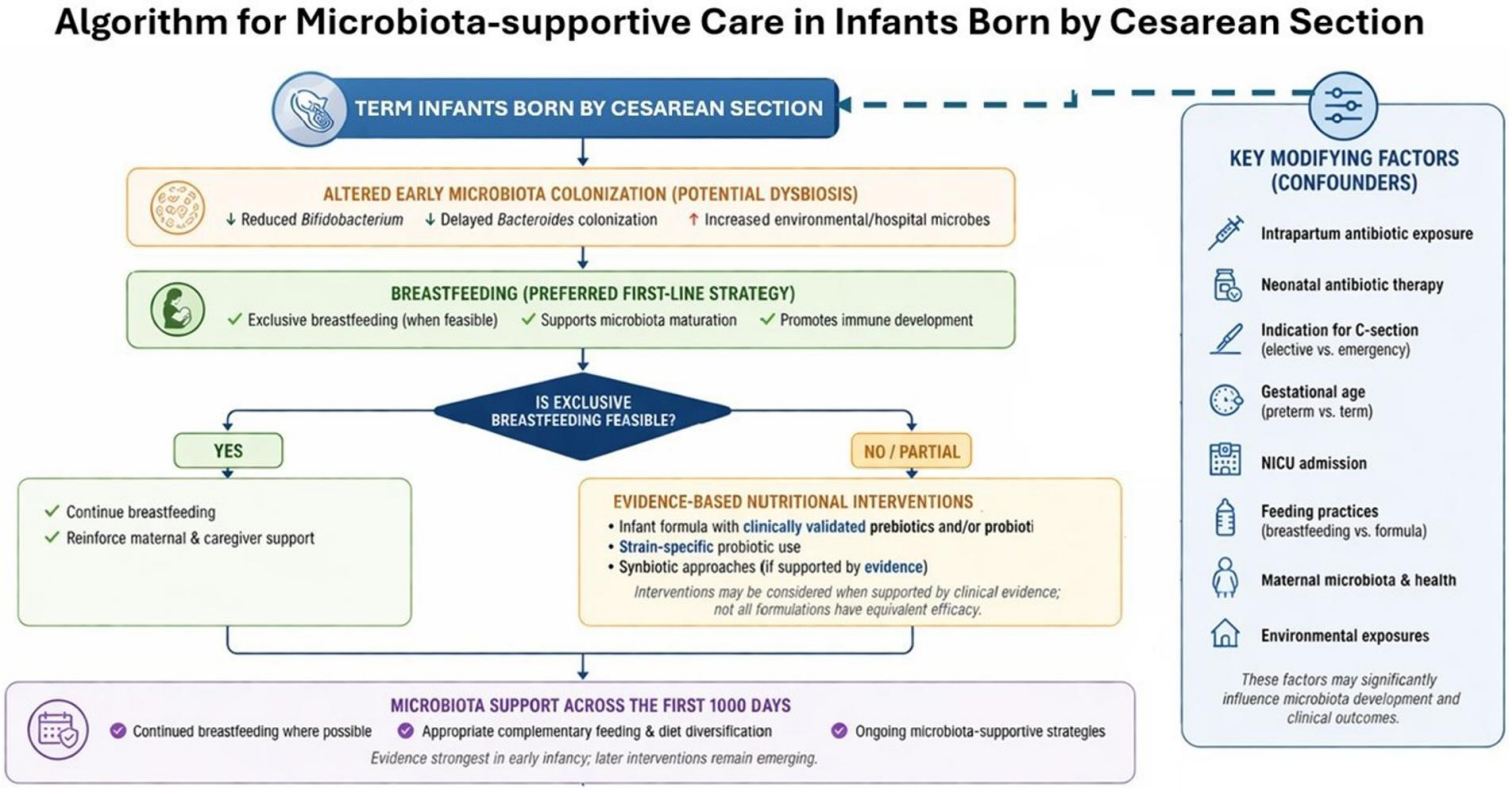
Clinical algorithm for microbiota-supportive care in term infants born by cesarean section, incorporating key modifying factors and evidence-based nutritional strategies across early life. This figure presents a structured clinical approach to supporting gut microbiota development in infants born by cesarean section. It outlines key steps from initial microbiota disruption risk through recommended interventions, with breastfeeding as the preferred first-line strategy. When exclusive breastfeeding is not feasible, evidence-based nutritional approaches, including selected prebiotic and probiotic interventions with demonstrated clinical benefit, may be considered. Importantly, microbiota development is influenced by multiple modifying factors, including intrapartum and neonatal antibiotic exposure, gestational age, indication for cesarean section (elective vs. emergency), neonatal care environment (e.g., NICU admission), feeding practices, and maternal health. These factors are explicitly incorporated into the algorithm to emphasize that infants should not be considered a homogeneous group. The algorithm reflects a continuity-of-care approach across the first 1,000 days of life; however, the evidence is strongest in early infancy. Associations between microbiota modulation and clinical outcomes are primarily derived from observational studies and should not be interpreted as causal.

In addition to guiding appropriate probiotic use, education must reinforce breastfeeding as the first and most effective strategy for promoting healthy gut colonization and immune development. While the manuscript's algorithm (Figure 2) includes breastfeeding, the narrative previously underrepresented its critical role as the primary intervention. The panel highlighted the need for consistent messaging to clinicians and caregivers that, whenever feasible, breastfeeding should be prioritized before considering alternative nutritional approaches. Moreover, educational efforts must extend beyond the immediate neonatal period and adopt a longitudinal perspective on gut health. The first 1,000 days—from conception through two years of age—represent a unique window of opportunity for microbiota modulation. Educating both providers and families about this extended timeline enables continued support for microbial health through breastfeeding, the introduction of solid foods, and evidence-based supplementation when appropriate (20, 67, 68).

Effective educational outreach must also address the practical gaps in implementation. The panel recommended that institutions and public health authorities develop updated clinical guidelines, continuing medical education (CME) programs, and culturally tailored educational resources tailored to regional healthcare settings. Such tools may help HCPs confidently differentiate between generic and evidence-based products, understand the significance of microbiota continuity, and counsel families more effectively. Ultimately, the panel concluded that education is not merely supportive; it is a core component of microbiota-informed care underpinning the effective implementation of clinical strategies and the sustainability of potential health benefits in early life.

## 4 Discussion

The rising global and regional prevalence of Cesarean section (C-section) delivery poses a growing challenge to infant health, particularly for the early development of the gut microbiota. Importantly, the impact of C-section on microbiota development is not uniform and is modified by several perinatal and postnatal factors, including antibiotic exposure, feeding mode, and early-life clinical conditions. This expert consensus confirms that infants born by cesarean section face a unique and potentially modifiable risk: disrupted microbial colonization in the neonatal period, often referred to as early-life dysbiosis. A substantial body of epidemiological and mechanistic research links these early microbial alterations to a range of later health outcomes, including allergic disease, immune-mediated conditions, metabolic disturbances, and functional gastrointestinal disorders (3, 12, 15, 17). It is important to emphasize that much of the available evidence linking C-section delivery to long-term health outcomes is derived from observational studies. Therefore, these associations should not be interpreted as causal, and residual confounding factors may contribute to the observed relationships (69).

The expert panel emphasized that addressing this challenge requires a multifaceted approach grounded in evidence and tailored to the specific vulnerabilities of infants born by cesarean section. Among all available strategies, breastfeeding remains the most effective and accessible intervention for promoting a diverse and beneficial gut microbiota, especially one rich in Bifidobacterium species (19, 20). Human milk provides critical immunological and prebiotic components that support microbial maturation, immune tolerance, and mucosal barrier function. The algorithm presented by the panel reiterates this recommendation: when possible, exclusive breastfeeding should be prioritized during the first 4–6 months of life, followed by continued breastfeeding alongside complementary feeding (Figure 2).

Vaginal seeding, defined as the transfer of maternal vaginal microbiota to infants born by cesarean section, has been proposed as a strategy to restore early microbial exposure. Initial studies demonstrated partial restoration of the microbiota following vaginal microbial transfer. More recent systematic reviews and randomized studies suggest potential benefits in microbial composition and immune outcomes; however, the evidence remains limited and heterogeneous. Importantly, major medical organizations currently caution against routine use of vaginal seeding due to potential risks of transmitting pathogenic organisms, including Group B *Streptococcus* and sexually transmitted infections. Therefore, this practice cannot currently be recommended outside of controlled research settings. Future research may explore safer alternatives, including defined microbial consortia or targeted probiotic strategies that mimic maternal microbial transfer (70–72).

When exclusive breastfeeding is not possible, the panel strongly recommends using formulas specifically designed and clinically demonstrated to restore microbial balance in infants born by cesarean section. These formulas should contain validated combinations of prebiotics and probiotics with documented efficacy—most notably the scGOS/lcFOS prebiotic blend in combination with *B. breve* M-16V. Evidence shows that this specific mixture supports early microbial colonization and mimics the quantity, diversity, and functionality of HMOs, thereby increasing SCFA production and modulating immune responses (21, 24, 26). Importantly, the panel stressed that not all probiotics are equally effective. Clinical practice must move beyond generalized recommendations and adopt a strain-specific mindset, where only interventions supported by data in infants born by cesarean section are considered appropriate. Misconceptions among healthcare professionals—such as assuming all probiotics offer the same benefit—can undermine the intended impact of such interventions (73, 74).

An important theme emerging from this consensus is the importance of maintaining microbiota support throughout the first 1,000 days of life, not just during infancy. While the first 1,000 days are widely recognized as a critical developmental window, the strength of evidence varies across this period. Robust data primarily support the neonatal and early infancy phases, during which microbiota establishment is most dynamic. Extensions of microbiota-targeted interventions beyond infancy are supported by emerging but still limited evidence and should therefore be interpreted as hypothesis-generating rather than conclusively evidence-based (75).

While most of the evidence presented focused on the neonatal and early infant period and did not include studies of maternal or post-infancy interventions specific to C-section populations, the panel recognized that microbiome development is a dynamic, long-term process. The gut microbiota remains highly adaptable during this early window, and opportunities to modulate microbial development persist well into toddlerhood (3, 25). Supporting gut health through consistent nutritional strategies, whether through breastfeeding, targeted formula use, or diet diversification, may yield long-term benefits in immunity, metabolism, and neurodevelopment. The first 1,000 days should therefore be viewed as a journey rather than a single intervention, and the continuity of microbial care must become a guiding principle in both clinical guidelines and parental counseling.

In this context, education emerged as a key enabler of effective implementation. As highlighted in the Results section, effective microbiota-informed care depends on HCPs and families having access to accurate, actionable, and evidence-based information. Addressing common knowledge gaps (e.g., that all probiotics confer similar benefits) is essential to ensure the appropriate use of evidence-based strategies, reinforce breastfeeding as the foundational intervention, and support informed decision-making regarding formula use and dietary transitions (67, 68). Public health messaging, CME programs, and culturally adapted family resources are all essential to ensure broad uptake and proper implementation.

Finally, the expert panel called for updated clinical guidelines and stronger alignment with policy. Many current protocols across institutions and countries do not adequately reflect the accumulating scientific evidence on microbiota development and its long-term health implications. Clinical inertia, logistical barriers, and knowledge gaps persist. Therefore, this consensus urges healthcare systems, research bodies, and industry partners to collaborate in translating microbiome science into real-world, culturally sensitive practice.

### 4.1 Limitations

While this expert consensus provides a comprehensive overview of the clinical implications of C-section delivery on infant gut microbiota and outlines evidence-based strategies for mitigation, several limitations must be acknowledged. First, although many of the recommendations are grounded in current clinical evidence and best practices, the long-term health outcomes associated with specific microbiota-targeted interventions—particularly in diverse populations—remain incompletely studied. Most existing studies focus on short- to medium-term endpoints, and robust longitudinal trials are still needed to evaluate the sustained impact of early-life microbiota modulation through nutritional strategies. Second, while the panel strongly supports the use of clinically validated probiotics and synbiotics, strain-specific data remain limited for some formulations. This underscores the need for more strain-targeted, high-quality randomized controlled trials (RCTs), especially in infants born by cesarean section from different geographic and ethnic backgrounds.

Third, despite the universal recommendation of breastfeeding as the gold standard for microbiota development, real-world barriers such as maternal health challenges, societal norms, and healthcare system limitations may limit its feasibility. The panel emphasizes that while alternative strategies can help mitigate dysbiosis, they are not substitutes for comprehensive lactation support and breastfeeding promotion. Furthermore, as with all consensus-based work, the conclusions and recommendations presented here reflect the collective expertise and interpretation of the selected panel members. Although a structured and transparent voting process was employed, there is an inherent risk of selection bias and variability in clinical experience across regions. Finally, although the expert panel included representatives from several Middle Eastern countries, the recommendations may not be fully generalizable to regions with different healthcare systems, population characteristics, or clinical practices. However, the biological principles underlying microbiota development are broadly applicable, supporting cautious extrapolation.

### 4.2 Recommendations and future research

This expert consensus highlights several actionable strategies to mitigate the adverse impact of C-section delivery on the infant gut microbiota. Foremost among these is the promotion and support of exclusive breastfeeding, which remains the gold standard for shaping a healthy microbiome during the first months of life. Exclusive breastfeeding should be encouraged for the first 4–6 months and continued up to two years, in alignment with global health recommendations. When breastfeeding is not feasible, healthcare providers are advised to recommend infant formulas specifically designed for infants born by cesarean section. These formulas should be enriched with clinically validated prebiotics and probiotics shown to promote colonization by beneficial microbes and restore microbial balance. A key recommendation is the preferential use of probiotic strains with proven efficacy in this context—such as *Bifidobacterium breve* M-16V—while avoiding non-specific or unsupported formulations, as depicted in the algorithm presented in Figure 2. In parallel, prebiotics should be selected based on demonstrated functional properties, appropriate composition, and clinical relevance. Importantly, not all prebiotics are equivalent; formulations such as scGOS/lcFOS have been shown in clinical studies to mimic the role of HMOs in infant nutrition. Education of both healthcare providers and parents is crucial to correct misconceptions, particularly the assumption that all probiotics are equally effective. Educational interventions should also emphasize the concept of microbiota continuity across the first 1,000 days and promote individualized nutritional strategies that adapt to the evolving needs of the growing infant.

Looking ahead, future research should address several important knowledge gaps. Longitudinal studies evaluating the sustained health outcomes of microbiota-targeted interventions—particularly regarding immune, metabolic, allergic, and neurodevelopmental endpoints—are urgently needed. Further, strain-specific clinical trials across diverse populations are required to strengthen the evidence base for probiotic use in infants born by cesarean section. Comparative effectiveness studies assessing different synbiotic blends vs. breastfeeding can offer additional insights into optimal nutritional strategies. Mechanistic research is also essential to better understand how early-life microbial exposures influence immune and metabolic programming. Finally, implementation science should explore real-world barriers to breastfeeding promotion, informed use of formula, and microbiota education, with the goal of improving clinical adherence and accessibility. By combining these recommendations with a focused research agenda, clinicians, researchers, and policymakers can better support the health of infants born by cesarean section and ensure that microbiota-informed care becomes an integral part of early-life health strategies.

## 5 Conclusion

In conclusion, this consensus supports a paradigm shift in early-life care for infants born by cesarean section that prioritizes breastfeeding, emphasizes evidence-based nutritional support, insists on synbiotic precision, and champions continuity across the first 1,000 days. Together, these strategies offer a powerful opportunity to mitigate the unintended effects of C-section delivery on infant gut health and to promote healthier outcomes across the lifespan.

## 6 Society endorsement

**“**Endorsed by the Saudi Society for Pediatric Gastroenterology, Hepatology & Nutrition (SASPGHAN)”.

## Data Availability

The original contributions presented in the study are included in the article material. Please direct any further inquiries to the corresponding author.
